# Editorial: Interaction between the vagus nerve and social communication

**DOI:** 10.3389/fnins.2025.1659288

**Published:** 2025-07-29

**Authors:** Rodrigo Daminello Raimundo, Luiz Carlos Marques Vanderlei, Moacir Fernandes Godoy, Vitor E. Valenti

**Affiliations:** ^1^Faculdade de Medicina do ABC, São Paulo, Brazil; ^2^Faculdade de Ciências e Tecnologia, Universidade Estadual Paulista (UNESP), São Paulo, Brazil; ^3^Faculdade de Medicina de São José do Rio Preto, São Paulo, Brazil; ^4^Faculdade de Filosofia e Ciências, Universidade Estadual Paulista (UNESP), São Paulo, Brazil

**Keywords:** vagus, communication, parasympathetic, ANS, HRV (heart rate variability)

The vagus nerve (cranial nerve X), as a central component of the parasympathetic nervous system, has long been recognized for its critical role in autonomic regulation. In recent years, its involvement in modulating social communication, emotional regulation, and even higher-order cognitive functions has emerged as a key area of interdisciplinary inquiry. The current Research Topic, *Interaction between the Vagus Nerve and Social Communication*, brings together novel experimental findings, clinical protocols, physiological assessments, and bibliometric analyses to highlight the multifaceted influence of vagal activity on health, behavior, and therapeutic potential.

The study by Collard et al. provides compelling experimental evidence from a rodent model, demonstrating that vagus nerve activity (VNA) undergoes gravity-dependent modulation during ictal events. This discovery offers an important step toward validating VNA as a potential biomarker for seizure detection and monitoring autonomic dysfunction in epilepsy. The implications are 2-fold: first, for improving the temporal precision of seizure prediction algorithms; and second, for integrating autonomic signals into real-time therapeutic feedback loops in neuromodulation strategies.

In the domain of prolonged disorders of consciousness (pDOC), Jiao et al. propose a timely and clinically relevant protocol using transcutaneous auricular vagus nerve stimulation (taVNS). Their methodological framework aims to stratify patients with pDOC based on electroencephalographic (EEG) features, identifying those most likely to benefit from taVNS. As the field of neuromodulation seeks to extend its reach into disorders of awareness, the emphasis on individualizing treatment based on neurophysiological criteria reflects a precision-medicine approach that holds great promise.

The contribution by Liu et al. complements the empirical and clinical perspectives with a comprehensive bibliometric analysis of VNS in the context of stroke rehabilitation. Their review charts the evolving landscape of research output, thematic clusters, and collaboration networks. The work not only highlights the growing scientific interest in VNS for post-stroke recovery but also offers a roadmap for future investigations, encouraging transdisciplinary integration and identifying underexplored frontiers in the field.

Kühne et al. turn our attention to the everyday psychophysiological correlates of social interaction. By analyzing heart rate variability (HRV) in a sample of healthy teachers during work and leisure days, the authors underscore the physiological impact of social stressors and occupational demands. Their findings reinforce the notion that social context and vagal tone are deeply intertwined, and that the quality of interpersonal interactions may shape autonomic regulation in meaningful ways.

Wang et al. explore the cognitive benefits and mechanisms of vagus nerve stimulation (VNS) in both healthy individuals and patients with neurological disorders. By synthesizing evidence from 100 studies, the authors highlight how VNS, particularly transcutaneous auricular VNS (taVNS), enhances emotional processing, executive attention, learning, and memory. Their findings underscore VNS's therapeutic potential in conditions such as epilepsy, depression, and Alzheimer's disease, and reveal how modulation of neural circuits and anti-inflammatory pathways may support cognitive regulation. These insights reinforce the role of VNS as a promising neurostimulation strategy for cognitive enhancement.

Taken together, these contributions illustrate the centrality of the vagus nerve as both mediator and modulator of social, cognitive, and physiological processes. Whether through its influence on seizure dynamics, its therapeutic engagement in consciousness disorders, its role in neurorehabilitation, or its subtle modulation of daily stress responses, the vagus nerve emerges in this Research Topic not merely as a conduit of parasympathetic tone, but as a dynamic interface between the body and social world.

We hope this Research Topic will serve as a catalyst for further interdisciplinary research, drawing connections between neuroscience, psychology, rehabilitation, cardiology, and social science. As we continue to unravel the bidirectional pathways of brain-body interaction, the vagus nerve will remain a focal point—both scientifically and clinically—at the crossroads of physiology and human connection.

